# Developing an AI Governance Framework for Safe and Responsible AI in Health Care Organizations: Protocol for a Multimethod Study

**DOI:** 10.2196/75702

**Published:** 2025-07-28

**Authors:** Sam Freeman, Amy Wang, Sudeep Saraf, Erica Potts, Amy McKimm, Enrico Coiera, Farah Magrabi

**Affiliations:** 1 Australian Institute of Health Innovation Macquarie University Sydney Australia; 2 Alfred Health Melbourne Australia

**Keywords:** artificial intelligence, governance, health care, oversight, patient safety, ethics, framework

## Abstract

**Background:**

Artificial intelligence (AI) has the potential to improve health care delivery through enhanced diagnostics, streamlined operations, and predictive analytics. However, health care organizations face substantial challenges in implementing AI safely and responsibly. This is due to regulatory complexity, ethical considerations, and a lack of practical governance frameworks. While many theoretical frameworks exist, few have been tested or adapted for real-world application in health care settings.

**Objective:**

This study aims to develop and validate a practical AI governance framework to support the safe and responsible use of AI in health care organizations. The specific objectives are to identify governance requirements for AI in health care, examine existing AI governance processes and best practices, codevelop an AI governance framework to meet the needs of health care organizations, and test and refine the framework through real-world application.

**Methods:**

A multimethod research design will be used, comprising four key stages: (1) a scoping review and document analysis to identify governance needs and current processes, (2) in-depth interviews with health care stakeholders as well as national and international AI governance experts, (3) development of a draft AI governance framework through a synthesis of findings, and (4) validation and refinement of the framework through stakeholder workshops and application to case studies of AI tools. Data will be analyzed using qualitative methods informed by grounded theory.

**Results:**

The project received funding in October 2023. Ethics approval was obtained from the Alfred Health Human Research Ethics Committee (project 171/24) and the Macquarie University Human Research Ethics Committee (project 16508). Data collection commenced in April 2024, with the scoping review and document analysis being finalized. As of March 2025, a total of 43 interviews have been completed. The final AI governance framework is expected to be completed and ready for dissemination by June 2025.

**Conclusions:**

This study will deliver a comprehensive AI governance framework co-designed with health care stakeholders to address real-world challenges in AI oversight. The framework will offer practical guidance to support health care organizations in adopting AI technologies safely, ethically, and in alignment with regulatory requirements. Outcomes from this study will inform local and international discussions on AI governance and promote the responsible integration of AI in health systems.

**International Registered Report Identifier (IRRID):**

DERR1-10.2196/75702

## Introduction

### Background

Artificial Intelligence (AI) technologies have the potential to transform health care by improving standards of care and addressing global challenges such as rising costs, staff shortages, and increasing patient complexity [1-4]. AI applications span both clinical and nonclinical tasks, including predicting patient care needs, assisting with diagnosis, and optimizing administrative processes [5]. Despite these benefits and technological advancements, adoption within health care organizations remains slow [6]. Compared to some other industries, health care is subject to significant regulation to ensure patient safety [7], and ethical considerations such as data privacy, transparency, and equity, which may limit adoption [8].

Governance at the organizational level is critical to ensure AI is implemented safely and responsibly. However, there is little practical guidance for governing AI development, implementation, and use in health care [9,10]. While numerous theoretical frameworks exist, these often focus on broad ethical principles such as fairness, transparency, and accountability but lack actionable strategies for health care organizations to adopt. This gap leaves organizations without clear mechanisms to assess AI risks, ensure compliance with regulatory standards, or integrate AI into clinical and operational workflows safely [8]. Most existing frameworks have not been designed for real-world application within complex health care environments. Few have been systematically tested or implemented, meaning there is limited evidence on their effectiveness in practice. Additionally, little is known about how key stakeholders such as health care professionals, patients, administrators, and AI developers perceive AI governance challenges or what governance mechanisms would best support responsible AI adoption [11]. Without a structured and adaptable governance framework, AI technologies may be deployed without sufficient oversight, leading to potential safety, ethical, and legal concerns.

Emerging evidence highlights safety concerns associated with AI and their impact on patient care, emphasizing the need for adequate governance to ensure a whole-of-system approach to safe AI implementation [12]. Effective governance at the organizational level is required to ensure that AI tools are effectively integrated into the IT infrastructure, as they are highly reliant on data from other clinical information systems [10]. For instance, data quality and requirements for any accompanying changes to the electronic medical record and other supporting clinical information systems need to be assessed to ensure data provided to the AI tools is fit for purpose and its output is accurately displayed to users. There is also a need to ensure that AI tools are integrated with the clinical workflow, with users aware of their intended use and trained to use them safely. Recent incidents of patient data being shared with the industry for AI training [13,14], along with evidence of hospitals deploying AI without adequate local evaluation for bias [15], also highlight the urgency for more stringent and consumer-centered AI governance.

The evolution of AI further necessitates a governance approach that can accommodate emerging technologies such as generative AI, AI-enabled clinical decision support systems, and automated administrative processes [16]. With the likelihood that AI capabilities will continue to progress, it is foreseeable that so will the potential risks, which will require governance approaches that are dynamic, iterative, and responsive to these technological and regulatory changes [17].

Health care must also consider organizational factors not addressed in broad conceptual frameworks, such as financial constraints, organizational culture, and commercialization [18]. Ongoing technological advancements and growing public awareness further necessitate governance strategies that adapt to emerging challenges. For example, the proliferation of generative AI, including AI scribes, has led to the development of new guidelines to address associated risks [19-21]. This study seeks to bridge these gaps by developing an AI governance framework tailored to the needs of health care delivery organizations, ensuring that AI innovations can be integrated safely and responsibly while maximizing their benefits for patient care and system efficiency [22].

### Aim

This study aims to develop and test an AI governance framework to enhance organizational oversight of AI applications in health care delivery.

### Objectives

To achieve the aim of the study, there are four specific objectives:

1. Identify governance requirements for AI applications in health care delivery organizations
2. Examine existing AI governance processes and current best practices for AI governance
3. Codevelop an AI governance framework to meet the needs of health care organizations
4. Test and refine the AI governance framework by applying it to exemplar AI tools

### Output

We will produce a comprehensive AI governance framework for a health care delivery organization.

## Methods

### Study Setting

This study will be conducted within a major Australian public health care organization that delivers care to a catchment of over 770,000 residents in Melbourne’s inner south. The organization operates across three hospital campuses, including a major tertiary and quaternary referral hospital that provides specialized care for patients who are critically ill. The other campuses focus on community-based health care, offering services in rehabilitation, geriatric medicine, general medicine, and aged mental health. Additionally, the organization delivers statewide and national health care services to patients across Victoria and Australia [23]. As a large and diverse health care provider, this setting offers a robust environment for developing and testing an AI governance framework for real-world application in health care. While the organization already has established AI-driven initiatives and governance structures supporting patient safety and digital health, there is currently no standardized AI governance framework tailored to its varied operational needs.

### Study Design

**Figure 1:**
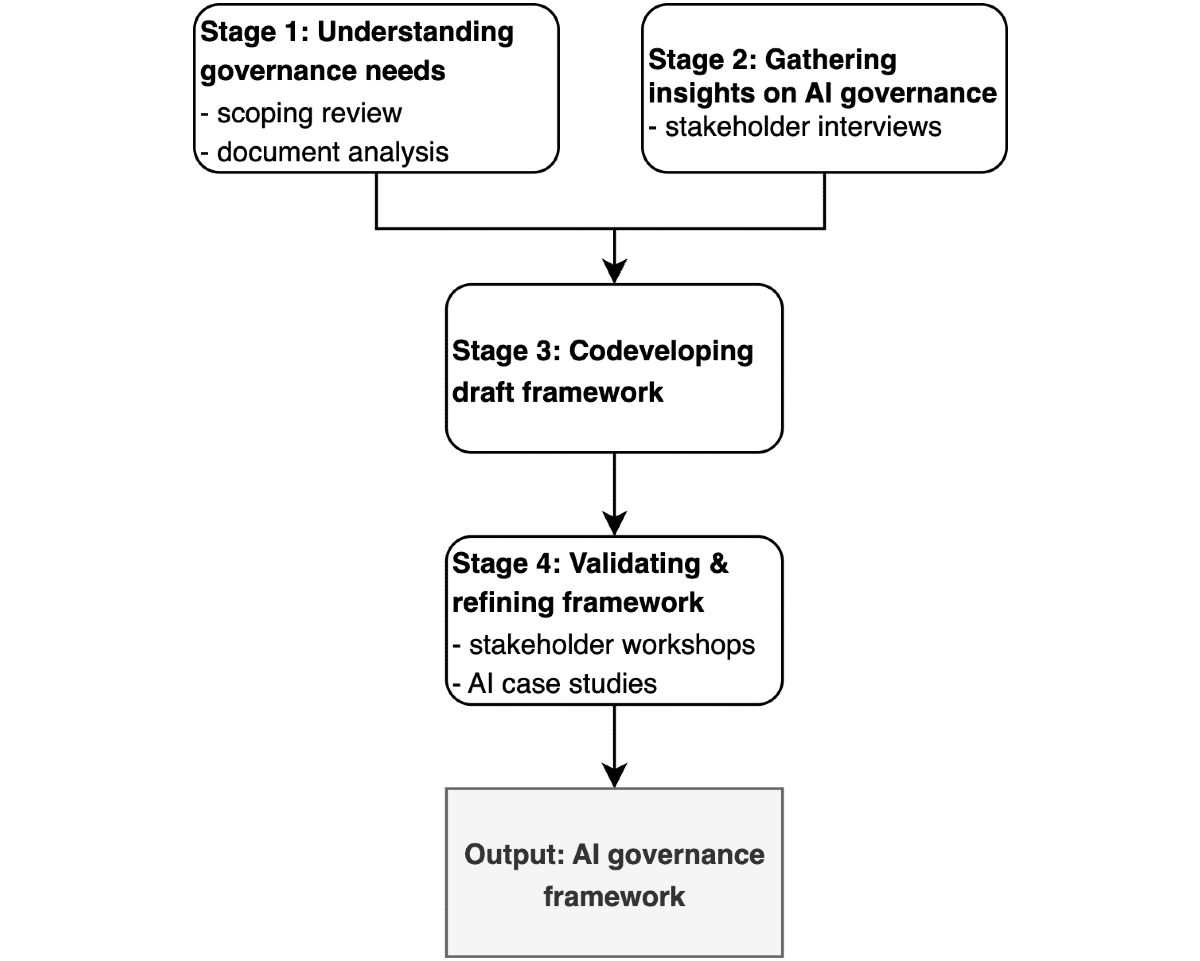
Study process diagram. AI: artificial intelligence.

We will adopt an exploratory multimethod approach [24] to develop and test an AI governance framework for the safe and responsible use of AI in health care delivery organizations. The structure of the study will comprise four stages that are directly linked to the study objectives: understanding governance needs through a scoping review and document analysis, gathering stakeholder insights on AI governance via in-depth interviews, codeveloping the AI governance framework through a synthesis of findings, and validating and refining the framework through stakeholder workshops and AI case studies (Figure 1).

Given the evolving nature of AI governance within health care organizations, we have developed a comprehensive research strategy that draws on existing methodologies for investigating emerging technologies [25,26], evaluating complex systems [27], and ensuring stakeholder engagement in governance development [28]. This qualitative study will be designed in accordance with COREQ (Consolidated Criteria for Reporting Qualitative Research) [29]. The final output of the study is to produce a practical and actionable AI governance framework that addresses key challenges in AI oversight at the organizational level. The research will run concurrently to enable insights from one stage to inform other stages iteratively. The four research stages are detailed in the following sections.

### Stage 1: Understanding Governance Needs

#### Scoping Review

Published literature about approaches to AI governance at an organizational level will be reviewed. Given the volume of literature published in this domain, only frameworks that are specific to health care will be reviewed [30]. The search strategy will be designed to address study site requirements, including governance approaches for AI tools with a clear intended purpose (eg, AI-enabled medical devices).

The following electronic databases will be searched: MEDLINE, Embase, and Scopus. These databases were selected based on consultation with a Macquarie University research librarian. A search will also be conducted on Google Scholar and Google search, where the first 200 results will be included for review. This is based on guidance around the use of Google for searching for gray literature [31]. Reference lists of key papers will be hand-searched for additional peer-reviewed and gray literature. Appropriate search strings will be developed by linking concept clusters relating to each component of the question (AI, governance and implementation, and a health care setting) with Boolean operators “AND” and “OR.” Search results will be imported into Covidence where a two-stage review process (title and abstract screening, followed by full-text review) will be conducted by two independent reviewers. Data will be extracted into Excel (Version 16.98; Microsoft Corporation).

#### Document Analysis

To ensure the AI governance framework aligns with the current state of governance processes at the study sites, a document analysis will be conducted to map current processes. This will involve a systematic review of relevant internal policies, governance documents, and procedural guidelines. This will help establish a baseline of governance structures to ensure that the AI framework does not replicate existing or previously established governance processes and to identify strengths and gaps in current governance approaches.

### Stage 2: Gathering Stakeholder Insights on AI Governance

#### Overview

In-depth semistructured interviews will be conducted with two key groups: health care staff and national and international experts in AI governance, policy, health care, AI, and patient safety. A purposive sampling technique will be used to identify key informants from both groups. A snowballing technique will also be used, allowing participants to refer other relevant informants for involvement in the study. Semistructured interview guides will be developed based on a review of the existing literature and input from the multidisciplinary project team. Members of the project team have recognized experience in health care, qualitative research, and AI safety and governance. Around 25-30 interviews (a total of 50-60) from both groups are expected to provide thematic saturation (where no new themes are identified). Key informants who agree to participate will be interviewed via online videoconferencing or in person, with audio recording. Audio files will be deidentified and professionally transcribed by a secure transcription service. Two investigators will independently analyze and code the interviews using open coding techniques based on grounded theory. A grounded theory approach will be applied as it provides a methodology and procedure for collecting and analyzing data and conceptualizing the results [32].

#### Health Care Stakeholder Interviews

We will recruit a diverse range of health care stakeholders to gain their perceptions on how to effectively govern the safe and responsible use of AI. The interviews will initially include informants from the board of directors and executive leadership, clinicians undertaking AI projects, clinical trial managers, and representatives from ethics and research governance offices, as well as clinical governance, risk, research, clinical operations, medical services, hospital liaison, legal, and consumer representatives. During the interviews, participants will be asked about current approaches to governance and processes to manage the safe and responsible application of AI in health care and barriers to adoption. In addition to health care stakeholder interviews, dedicated consumer consultation will be incorporated into the framework’s development process. To do this, we will engage the Australian Institute of Health Innovation Consumer Engagement Panel, which supports person-centered research. The panel will provide their perspectives on governance priorities through review sessions. Feedback from these sessions will be analyzed using a grounded theory coding approach and integrated alongside other stakeholder data to inform the development of the AI governance framework.

#### National and International Expert Interviews

We will examine current practices for governing AI in health care. Experts will include participants from academia (ethics, governance, digital health, technology, AI), government, health care administration, clinical settings, and professional health care associations. Participants will be asked about current approaches to AI governance in health care and will be invited to share any documentation about governing AI as well as tools or templates used to support AI governance processes.

### Stage 3: Codeveloping a Draft AI Governance Framework

Findings from the scoping review, document analysis, and stakeholder interviews will be synthesized to develop a draft AI governance framework tailored to the needs of health care organizations. A grounded theory approach will guide the synthesis of findings across the scoping review, document analysis, and stakeholder interviews. Data from each source will be open coded to identify key concepts, which will then be compared and integrated through constant comparative analysis [33]. Emerging categories will be iteratively refined to develop the framework structure to reflect insights from stages 1 and 2.

The framework will outline pathways for integrating AI governance within existing clinical and operational workflows. To do this, the draft framework components will be mapped against current clinical governance, digital health, risk management, quality improvement processes, etc. Here, potential points of alignment and areas requiring modification will be identified. To address challenges such as organizational resistance, workflow incompatibilities, and training needs, the framework will include implementation recommendations outlining strategies for change management, staff education, and stakeholder engagement, informed by stakeholder insights.

During this phase, the framework will be designed to apply to different types of AI applications, including emerging technologies such as large language models and generative AI. A stratified governance approach based on the type of AI application and risk profile will be considered to address common and emerging risks, such as explainability challenges, hallucinations, etc, where identified. Additionally, specific governance components will be developed based on study findings to ensure the framework remains applicable across evolving AI technologies. The draft AI governance framework developed in this stage will be tested and validated in stage 4 through stakeholder workshops and AI case studies.

### Stage 4: Validating and Refining the Draft AI Governance Framework

#### Stakeholder Workshops

Stakeholder workshops will be conducted to refine and validate the AI governance framework with key stakeholders from the health care organization. Stakeholder workshops will be structured using facilitated discussion and scenario-based exercises focused on the practical application of the draft framework. The number of workshops and participants will be determined in consultation with the study site’s project team to ensure appropriate representation of clinical, operational, and governance stakeholders. Detailed notes will be taken during the workshops, which will be analyzed using a grounded theory approach to identify key themes and areas for framework refinement. Insights from the workshops will be incorporated iteratively to improve the framework’s practicality, ease of use, and alignment with organizational needs.

#### Testing the Draft Framework on Case Studies

The draft governance framework will be applied to exemplar AI tools from the study site to determine whether it provides adequate oversight. We will select 3-5 AI tools that are representative of the different pathways by which health care organizations acquire and use AI tools, including in-house development, codevelopment with external partners, and procurement of external AI tools. Selection criteria for case studies will also include representation of both clinical and administrative applications, diversity in levels of autonomy (eg, assistive vs decision support) [1], and variation in associated risk profiles. Tools will be selected in consultation with the study site’s project team to ensure relevance and alignment with the organization’s operational and clinical priorities. To evaluate the framework’s usability and adaptability, a think aloud protocol will be used during pilot testing [34]. Participants will talk through their experience as they apply the framework to the AI tool in real time, providing insights into its effectiveness and identifying areas for improvement in the checklist. These findings will inform iterative refinement of the framework to enhance its applicability across diverse implementation contexts.

We will also assess the extent to which the AI framework will continue to be fit for purpose for current and next-generation AI technologies. Findings from these case studies and stages 1-3 will help identify gaps to refine the AI governance framework. The framework will be evaluated during this study based on its perceived usability, completeness, relevance to risk management and regulatory requirements, and stakeholder acceptance. This will be assessed with feedback collected during the stakeholder workshops and case study pilots.

## Results

This study received funding in October 2023 from the Digital Health Cooperative Research Centre, Alfred Health, and Macquarie University. Ethics approval was granted by the Macquarie University Human Research Ethics Committee (project 16508) in December 2023 and Alfred Health Human Research Ethics Committee (project 171/24) in February 2024. Data collection commenced in April 2024 and is progressing as planned.

As of March 2025, the scoping review and document analysis (stage 1) are underway, with preliminary findings identifying key themes in existing AI governance frameworks and internal governance structures at the study site. Interviews with health care stakeholders and AI governance experts (stage 2) are complete (n=43), and the thematic analysis has commenced. Case study selection for framework testing has been finalized in consultation with the study site. The final AI governance framework is anticipated to be finalized and disseminated by June 2025.

## Discussion

### Study Significance and Strengths

This study will generate critical insights into AI governance by developing and validating a framework for the safe and responsible integration of AI in health care. By addressing the gap in practical guidance for governance at the health care organizational level, this study will provide actionable strategies for managing AI tools in both research and operations. Its multimethod approach will ensure a comprehensive understanding of AI governance by integrating findings from a scoping review, stakeholder engagement, and case studies. This methodology will support the development of a governance framework that is both theoretically grounded and practically applicable.

A key strength of this study is the inclusion of diverse stakeholder perspectives, ensuring that governance recommendations are aligned with real-world requirements. By systematically developing an AI governance framework, this study will provide evidence-based recommendations for improving oversight of AI tools in health care delivery. It will ensure that organizational policy supports safe and responsible AI and complies with legislation (eg, data privacy, consumer law, and cybersecurity) and relevant AI ethics frameworks. The findings will contribute to international discussions on AI governance and support the development of governance that balances adaptability with patient safety and ethical considerations.

### Limitations

Despite the strengths of this study, its limitations should be acknowledged. The study is currently limited to one Australian health care organization, which may limit the generalizability of the AI governance framework to other settings with different regulatory environments, organizational structures, or levels of technical maturity. While stakeholder interviews will include international participants, the findings may still reflect context-specific challenges rather than universal governance solutions. This also means that the successful integration of the framework into existing structures and organizational cultures may vary depending on local governance systems, staff engagement, and available training resources. Furthermore, while the framework is being developed at a large metropolitan health care organization, limiting generalizability, it is being designed to be adaptable to support its application in diverse health care settings (eg, regional hospitals, resource-limited settings). However, further research will be needed to evaluate the framework’s scalability and effectiveness across diverse health care environments.

Additionally, the study focuses on governance at the health care organizational level, which may not account for broader policy, legislative, or technological changes that might impact AI adoption in health care. The rapid progress of AI technologies such as generative AI presents challenges in ensuring the framework remains adaptable and relevant over time. While the iterative design of this study allows for ongoing updates, future research will be needed to assess the long-term sustainability and adaptability of the framework as AI capabilities continue to evolve.

### Future Directions

This study will provide an AI governance framework specifically designed for health care organizations; however, further research will be needed to ensure its long-term applicability and scalability within dynamic health care contexts. Future studies should focus on implementing and evaluating the framework’s effectiveness across a broader range of health care organizations, including regional and rural health services, to assess its adaptability in diverse settings. Expanding the research to include international case studies will also provide comparative insights into how different regulatory and health care systems approach AI governance. Further research, including longitudinal follow-up studies, will be critical to track the sustained impact of AI governance frameworks on patient safety, operational efficiency, and clinician trust over time. This includes researching how governance processes develop alongside emerging AI technologies and regulatory changes as well as the long-term effectiveness and sustainability of the AI governance framework itself. This research should evaluate real-world outcomes such as long-term adoption, compliance improvement, and risk reduction.

### Conclusions

This study will develop and validate an AI governance framework to support the safe and responsible use of AI in health care. By integrating a multimethod approach, the study will provide practical guidance on AI oversight at the organizational level, addressing gaps in existing governance structures. Findings will contribute to international discussions on AI governance, ensuring that AI technologies are adopted in a way that prioritizes patient safety, ethical considerations, and regulatory compliance. The iterative nature of this research will enable the framework to adapt to evolving AI capabilities and organizational needs, providing a foundation for robust governance strategies in health care.

## Abbreviations

AI: artificial intelligence
COREQ: Consolidated Criteria for Reporting Qualitative Research

## Data Availability

The datasets generated during this study will not be made publicly available for privacy reasons, but requests to the corresponding author will be considered on a case-by-case basis.
